# Activated Carbon Modification towards Efficient Catalyst for High Value-Added Products Synthesis from Alpha-Pinene

**DOI:** 10.3390/ma14247811

**Published:** 2021-12-17

**Authors:** Joanna Sreńscek-Nazzal, Adrianna Kamińska, Piotr Miądlicki, Agnieszka Wróblewska, Karolina Kiełbasa, Rafał Jan Wróbel, Jarosław Serafin, Beata Michalkiewicz

**Affiliations:** 1 Department of Catalytic and Sorbent Materials Engineering, Faculty of Chemical Technology and Engineering, West Pomeranian University of Technology in Szczecin, Piastów Ave. 42, 71-065 Szczecin, Poland; kaminska.adrianna@zut.edu.pl (A.K.); piotr.miadlicki@zut.edu.pl (P.M.); Karolina.Kielbasa@zut.edu.pl (K.K.); rafal.wrobel@zut.edu.pl (R.J.W.); Beata.Michalkiewicz@zut.edu.pl (B.M.); 2 Barcelona Research Center in Multiscale Science and Engineering, Department of Chemical Engineering, Institute of Energy Technologies, Technical University of Catalonia, Eduard Maristany 16, 08019 Barcelona, Spain; jaroslaw.serafin@upc.edu

**Keywords:** alpha-pinene isomerization, commercial activated carbon, acid treatment

## Abstract

DT0-activated carbons modified with HCl and HNO_3_ acids, which were used for the first time in the catalytic process of alpha-pinene isomerization, are presented in this study. The carbon materials DT0, DT0_HCl, DT0_HNO_3_, and DT0_HCl_HNO_3_ were examined with the following methods: XRF, SEM, EDX, XPS, FT-IR, XRD, and N_2_ adsorption at −196 °C. It was shown that DT0_HCl_HNO_3_-activated carbon was the most active material in the alpha-pinene isomerization process. Detailed studies of alpha-pinene isomerization were carried out over this carbon by changing the reaction parameters such as time (5–180 min) and temperature (60–175 °C). The 100% conversion of alpha-pinene was achieved at the temperature of 160 °C and catalyst content of 5 wt% after 3 h over the DT0_HCl_HNO_3_ catalyst. Camphene and limonene were the main products of the alpha-pinene isomerization reaction.

## 1. Introduction

In recent years, there has been a growing interest in commercial, porous, activated carbons as materials that can be used in many fields of research. Due to the developed specific surface and porosity, activated carbons belong to the group of materials of various applications and constantly growing practical importance.

Currently, activated carbons (AC) are obtained from raw materials rich in elemental carbon, which include waste materials from industry and agriculture or alternative materials. Materials that belong to the first group and are used on an industrial scale include coconut shell [1] and nutshell [2].

Another group are alternative materials that are used in the production of commercial activated carbons. This group includes, among others: charcoal [3], peat [4,5,6] anthracite [7,8,9] wood [10,11,12], and municipal solid waste [13]. The content of elemental carbon in agricultural and industrial waste is lower than in the alternative materials [14]; therefore, the efficiency of the production of activated carbons from waste materials is lower than in the case of AC production from alternative materials [15].

Activated carbons are characterized by a well-developed porous structure and a large specific surface area as well as varying surface chemistry. The chemical properties of carbonaceous materials are attributed to the presence of various functional groups and heteroatoms (oxygen, nitrogen, hydrogen, sulfur, potassium, sodium, aluminum, and phosphorus) [16]. The presence of these atoms in the structure of activated carbons may result from their introduction into the carbon matrix during precursor activation/post-synthesis modification or may result from the content of these elements in the raw material used [17,18].

Modification is a promising and effective way to increase the functionality of carbonaceous materials, which can then be used in many fields [19,20]. In order to improve the chemical properties, the surface modification of activated carbons is used. Surface modification can be divided into thermal modification [21] and chemical modification [22]. In addition, there are reports in the literature on the biological modification of activated carbons (using bacteria) [23,24].

Chemical modification, in turn, is divided into liquid and gas modification [25]. The most widely studied compounds used for liquid modification are nitric acid [26], sulfuric acid [27,28] phosphoric acid [28,29], hydrochloric acid [30], and hydrogen peroxide [31].

The modification of activated carbons with acids allows for the oxidation of their surface and an increase in the hydrophilicity of the surface of the carbonaceous material. In addition, during acid treatment, non-organic and ash residues are removed from the material. This process also increases the acidic properties of the activated carbons that have been treated with the acid [32].

In the work presented by Aggarwal et al., granulated activated carbon was subjected to a liquid-phase oxidation process in the presence of nitric acid, ammonium persulphate, and hydrogen peroxide. Modification with these compounds increased the number of oxygen groups on the surface of the tested materials and thus allowed for the increase of the functionality of the granulated activated carbons [33].

Another team used HNO_3_ to modify activated carbons. It was reported that the oxygen functional groups that were mainly formed were carboxylic acid, phenolic, lactone, and quinol groups. The presented results clearly indicated the relationship between the presence of oxygen functional groups and the activity of active carbons in the tested process. It was concluded that, along with the increase in the number of functional groups on the surface of activated carbon, its sorption capacity increased [34].

In the work presented by the team of Strelko [35], commercial activated carbon was treated with nitric acid in order to introduce acid functional groups into the material. The acid treatment resulted in a decrease in the surface area and pore volume and at the same time, the modified carbon was characterized by an acid surface (the dominant surface functional groups were carboxyl groups). After the modification, the sample showed cation-exchange activity in a wide pH range; thus, it was more active in the investigated metal adsorption process.

Activated carbon was also modified with HCl and NaOH solutions [36]. The carbonaceous material exposed to HCl and KOH was characterized by the presence of (carboxyl, hydroxyl, and carbonyl) functional groups. Chemical treatment changed the surface of functional groups, surface morphology, and texture of the activated carbon. Modification with HCl and NaOH increased the adsorption selectivity of the tested activated carbon.

Wu et al. [30] used HCl, HNO_3_, and NaOH as compounds to modify the surface of activated carbon. It was noticed that the number of C-O functional groups increased for the carbons subjected to acid modification, while their number decreased after alkaline modification. By means of chemical activation, the modified activated carbon showed a higher adsorption efficiency of organic chemicals.

Xu et al. [37] used HNO_3_ in various concentrations to modify the surface of the activated carbon. For activated carbons after modification, an increase in the number of carboxyl groups was observed as compared to the untreated activated carbon. As the concentration of the HNO_3_ solution increased (up to 20% of HNO_3_ solutions), the amount of functional groups on the AC surface also increased and then decreased (50% of HNO_3_ solution). By chemically modifying the surface, this carbon became more efficient in the ion Pb^2+^ adsorption process.

Activated carbons subjected to surface modification with acids have found application in many chemical processes. They have been widely used to adsorb many chemical compounds, including azo dyes [38], acid dyes [39], mercury [40], benzene and toluene [41], VOC (volatile organic compounds) [42], lead [43], chromium [44], and manganese ions [45]. Modified activated carbons have also been used in electrochemistry as supercapacitors [46,47,48] and as capacitive and pseudocapacitive energy storage devices in supercapacitors [49]. In catalysis, acid-modified activated carbons have been used as catalysts for acetylation of glycerol [50], in oxidative carbonylation of methanol [51], esterification of fatty acids [52], and the synthesis of N-alkylimidazoles and imidazolium ionic liquids [53].

Alpha-pinene belongs to the group of monoterpenes with the general formula C_10_H_16_. Taking into account the fact that this compound has a double bond between carbon atoms, it can be transformed into many useful compounds. These compounds can be used in many industries [54].

The main products of alpha-pinene isomerization are camphene and limonene. The first is a bicyclic monoterpene with a pleasant camphor scent. Camphene is characterized by strong aromatic properties, thanks to which it has been used in many industries. In the perfume industry, camphene is used as one of the fragrance components. The use of essential oils based on camphene in the food industry is aimed at improving the smell and deepening the taste of dishes [55]. Additionally, thanks to its fragrance properties, camphene has a repellent effect against various insects [56].

Limonene belongs to single-ring monoterpenes with a characteristic citrus scent. Limonene can be obtained by steam distillation from the peel of citrus fruits [57]. In the food industry, this compound is used as a flavoring additive to improve the taste and aroma of dishes. In the cosmetics industry, limonene is used in the production of cosmetics [58]. Limonene has also been used as a bio-solvent in the household chemistry industry [59] and as a natural and ecological repellant [60].

The isomerization of alpha-pinene on an industrial scale is carried out with TiO_2_ acidic catalyst, and the temperature of the process ranges from 150 to 170 °C. One of the disadvantages of this method is the fact that the catalyst must be synthesized “in situ” because it is not commercially available. Moreover, the reaction rate is slow [61], and the selectivity to bicyclic and monocyclic terpenes is insufficient [62].

At present, the aim is to use catalysts that show higher activity and selectivity for the main products of alpha-pinene isomerization. The catalysts that have been used in the alpha-pinene isomerization reaction are zeolites [63], silica [64,65], ion exchange resin [55], H-mordenite molecular sieves [62], 2D Ti_3_C_2_TxMXene [66], zirconium sulfate [67], activated TiO_2_ [68], and natural aluminosilicates modified with acid [69].

This paper presents the process of alpha-pinene isomerization over commercial DT0 catalysts modified with hydrochloric and nitric acids. To the best of our knowledge, such catalysts have not been used in the alpha-pinene isomerization up to now. In these studies, we used commercial carbon DT0, which was modified in a different way than in our previous work [70], in which activated carbon (EuroPh and FPV) was modified with plasma. An advantage of the acid-modified DT0 catalyst was that it was obtained with a simple, one-step, low-cost method compared to the expensive activated carbon plasma modification method. Our research aimed to compare the activity of modified DT0 catalysts under specified conditions of alpha-pinene isomerization. The influence of temperature on the rate of alpha-pinene isomerization was investigated for the most active catalyst in the reaction. The catalytic activity was determined by the alpha-pinene conversion values and the selectivity of limonene and camphene. The conducted studies also provided significant information related to the physicochemical characteristics of the DT0 modified carbons.

## 2. Materials and Methods

### 2.1. Chemicals and Materials

Commercial activated carbon DT0 with diameter grain 0.5 mm (Grand Activated, Hajnówka, Poland) was selected as the starting material for preparing catalysts for the alpha-pinene isomerization process. Hydrochloric acid (35%–38% p.a., CHEMPUR, Piekary Śląskie, Poland) and nitric acid (65% p.a., CHEMPUR, Piekary Śląskie, Poland) were used to treat ACs. The acid-treated ACs will be referred to by their name and type of acid used for treatment. Samples with commercial activated carbon treated with HCl will be named DT0_HCl, with HNO_3_—DT0_HNO_3_, and with both—DT0_HCl_HNO_3_.

#### Modification of ACs with Acids

Acs were treated with hydrochloric and nitric acids. A commercial activated carbon was continuously modified in different ways: first with hydrochloric acid; second with nitric acid; and for the last one, two-step modification was used and, finally, a two-step process (firstly with hydrochloric and then nitric acid) to prepare modified ACs with different acid groups. First, 10 g of activated carbon named DT0 was ultrasonically washed with deionized water to remove small activated carbon powder. The sample was poured into a round bottom flask and placed in a heating mantle (CHEMLAND, Stargard, Poland). Then 100 mL of hydrochloric acid (at an acid to DT0 ratio of 1:1) was added into a round bottom flask, and the obtained mixture was refluxed. AC modification was continued for 2 h. Finally, activated carbon was washed repeatedly with deionized water until pH was constant and dried at 120 °C overnight. The same procedure was used with nitric acid.

### 2.2. Textural Properties and Chemical Characterization of the Carbons

The textural parameters of the modified ACs were investigated with N_2_ adsorption/desorption at −196 °C using an ASAP 2460 instrument (Micrometrics, Norcross, GA, USA). The samples were degassed at 250 °C in a vacuum environment for 14 h before measurements. Experimental adsorption data at a relative pressure (P/P0) of less than 0.3 were used to calculate surface area values using the standard Brunauer, Emmett, and Teller (BET) equation. The pore size distribution was determined using the density functional theory (DFT) based on nitrogen adsorption data. For the DFT method, the N_2_-DFT adsorption slit model was used.

The elemental composition of modified catalysts was examined using the energy dispersive X-ray fluorescence (ED-XRF) (Epsilon 3, PanAnaltical, Almelo, The Netherlands). The measurement was performed in a helium atmosphere using the OMNIAN standardless analysis program.

The morphology of the samples was observed via scanning electron microscopy with cold emission (SU8020, Ultra-High Resolution Field Emission Scanning Electron Microscope; Hitachi Ltd., Hitachi City, Japan). Images were taken with a 5 kV accelerating voltage using a triple detector system. The microscope was additionally equipped with an Energy Dispersive Spectroscopy system (EDS; EDSNSS312, Thermo Fisher Scientific, Waltham, MA, USA), owing to which qualitative analysis could be performed.

FTIR spectra of the modified carbon samples were obtained utilizing a Nicolet 380 ATR-FTIR spectrometer (Thermo Fisher Scientific Inc., Waltham, MA, USA). Before the spectrum of a sample was recorded, the background line obtained was arbitrarily and automatically subtracted. The spectra were recorded in the range of 4000–400 cm^−1^.

The X-ray photoelectron spectroscopy measurements were performed in a commercial multipurpose (XPS, LEED, UPS, AES) UHV surface analysis system (PREVAC) (PREVAC, Rogów, Poland). The routinely obtained base pressure was in the low 10^−10^ mbar range. The analysis chamber was equipped with a non-monochromatic X-ray photoelectron spectroscopy (XPS, PREVAC, Rogów, Poland) and kinetic electron energy analyzer (SES-2002; Scienta Scientific AB, Uppsala, Sweden). The calibration of the spectrometer was performed using Ag 3d5/2 transition. The pulverized samples were thoroughly degassed under vacuum prior to measurement. During XPS measurements, the vacuum was in the low 10^−9^ mbar range. The X-ray Mg K_a_ (hn = 1253.7 eV) radiation was applied.

Powder X-ray diffraction was used for the analysis of the AC structure by measuring the intensity of radiation reflected at various angles (X’Pert–PRO, Panalytical, Almelo, The Netherlands). Copper radiation (Kα1 = 0.154056 nm) was used. Measurements were performed for the range of 10~100 in 2θ scale for modified ACs. The performed diffractograms were analyzed by comparing the position and intensity of the reflections on the obtained diffractograms with the standard diffractograms from the ICDD PDF4+2015 database based on the X’Pert HighScore computer program.

### 2.3. Alpha-Pinene Isomerization Method

Studies on the activity of DT0 materials in α-pinene isomerization process were carried out in a 10 cm^3^ glass reactor equipped with a reflux condenser and magnetic stirrer with a heating function. For studies on isomerization, 2 g of α-pinene (98%, Aldrich, Sigma-Aldrich, Burlington, MA, USA) and the appropriate amount of catalyst were weighed into the reactor, which was then placed in an oil bath. The mixing speed was constant at 500 rpm. The effect of the following parameters was examined: temperatures (in the range of 145–175 °C) and reaction time (from 5 to 180 min).

Quantitative analyses were performed with a Thermo Electron FOCUS chromatograph (Waltham, MA, USA) equipped with an FID detector and a ZB-1701 column (30 m × 0.53 mm × 1 um). The parameters of the analyses were as follows: helium flow of 1 mL/min, sample chamber temperature of 220 °C, detector temperature of 250 °C, the temperature of the furnace—isothermally for 2 min at the temperature of 50 °C followed by growth step of 5 °C/min to 100 °C, and growth with the increment of 10 °C/min to 200 °C. In order to determine the composition of the post-reaction mixtures, the internal normalization was used.

## 3. Results and Discussion

### 3.1. Characterization

**Figure 1:**
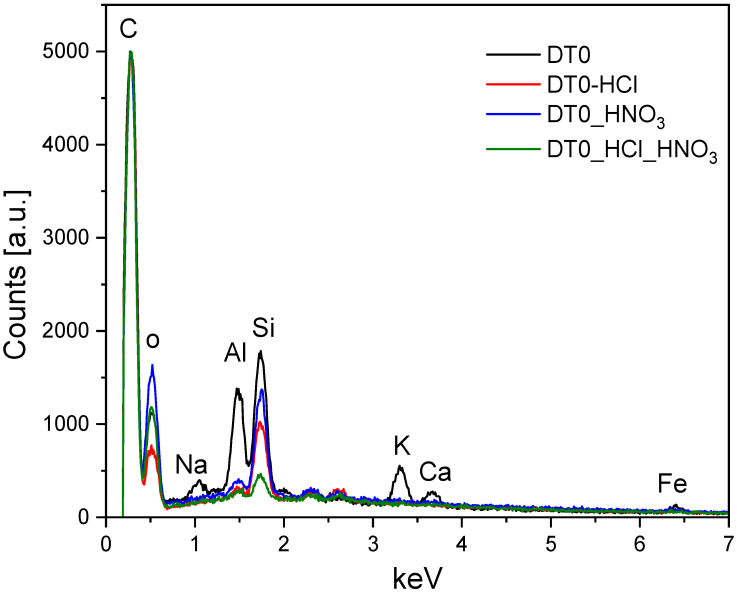
EDX spectra of DT0 samples.

Figure 1 shows a SEM-EDX image of typical DT0 samples before and after modification.

The EDX analysis revealed that the pristine sample contained, besides carbon, the following elements: oxygen, sodium, alumina, silicon, potassium, calcium, and iron. After acid treatment, Na, K, Ca, and Fe were completely removed. Al and Si were partially removed. The quantitative results of EDX measurements are presented in Table 1. The EDX analysis was confirmed by XRF measurements. X-ray fluorescence spectroscopy (XRF) is a powerful analytical technique that provides both qualitative and quantitative information on a wide variety of sample types including solids, liquids, slurries, and loose powders. Theoretically, it can quantify elements from sodium up to uranium. Practically the detection of small amounts of sodium is difficult. This is the reason that there is no carbon, oxygen, and sodium in Table 2 concerning elemental analysis based on XRF measurements. These elements are listed in Table 1—with the elemental analysis on the basis of EDX measurements. The quantitative results of XRD and XRF results were comparable, and these different techniques allowed us to draw the same conclusions. The elements present in DT0 were completely or partially removed after acid treatment.

According to XRF measurements, alumina was completely removed after acid treatment. However, EDX investigation show the presence of Al below 1%. Alumina is less soluble in acids than Na, K, Ca, and Fe. It is well known that the solubility of silicon in acids is very low, but we were not able to identify silicon compounds if they were amorphous. Thus, we can say that the silicon and its compounds present in DT0 were very difficult to dissolve in acid, which came as no surprise.

**Figure 2:**
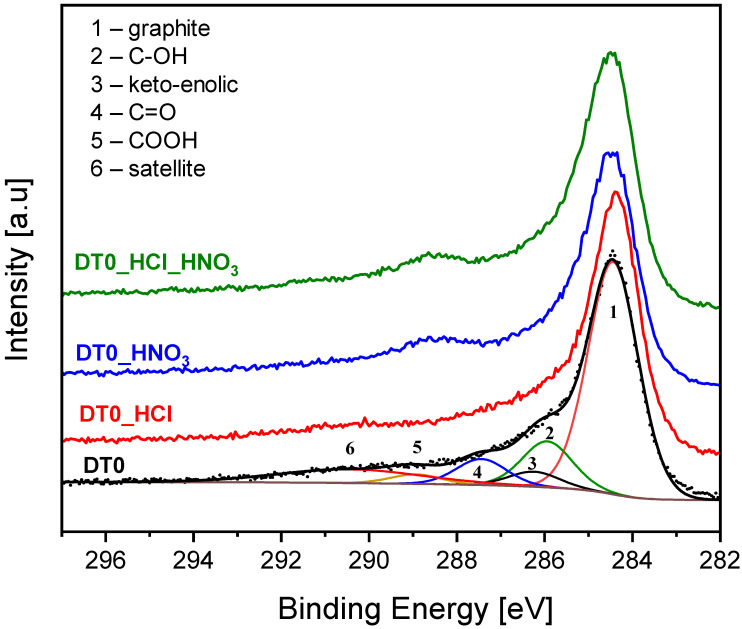
X-ray photoelectron C1s spectra. For the DT0 the components are indicated.

The XPS analysis enables quantitative determination of functional carbon groups [71]. Depending on the kind of bonding and amount of oxygen atoms attached the shift of carbon signals towards higher binding energies can be observed. The C1s signals presented in Figure 2 were carefully deconvoluted to components according to the procedure given elsewhere [72]. Table 3 presents the elemental content of the surface expressed as atomic concentration.

The tail on the left-hand side of the dominant component is related with the presence of carbon-oxygen species. In DT0_HNO_3_ and DT0_HCl_HNO_3_ samples a hump at about 289 eV is observed. This can be attributed to a significant increase of COOH groups over the carbon surface. It is clearly the oxidation effect of HNO_3_. Table 4 presents surface elemental contents of the samples.

**Figure 3:**
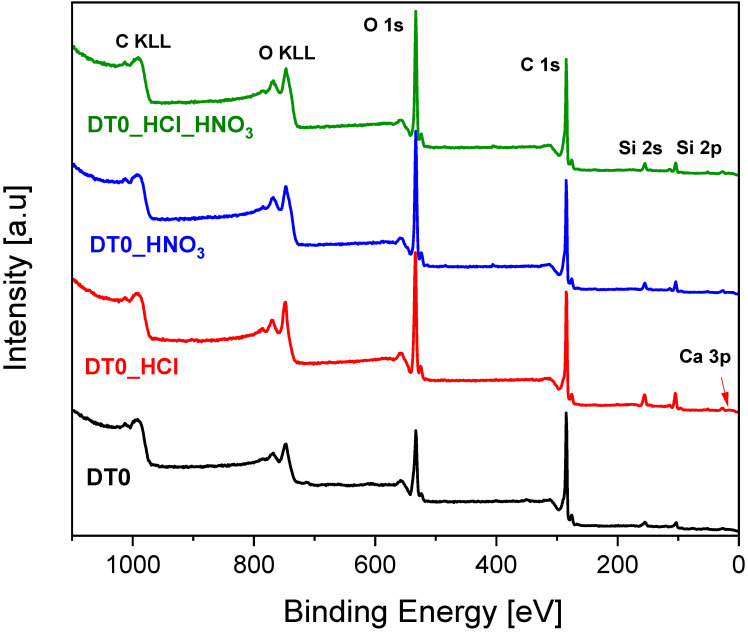
X-ray photoelectron survey spectra.

Figure 3 presents the XP spectra of the analyzed samples.

The content of calcium decreases as a result of acid treatment. The higher content of silicon after acid treatment is most probably the effect of removing the calcium screening effect. In the case of DT0_HCl sample, the increase of oxygen content is observed. This is not the result of oxidation but rather an effect of calcium removal as HCl is not an oxidizing agent. Such effects were observed previously [72].

**Figure 4:**
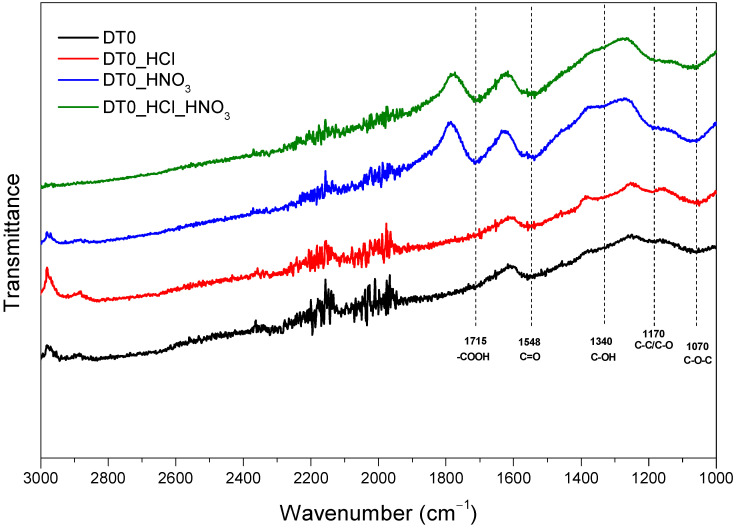
FTIR spectra of DT0, DT0_HCl, DT0_HNO_3_, and DT0_HCl_HNO_3_.

Figure 4 shows the FTIR spectra of activated carbons.

Figure 4 shows FTIR spectra of DT0 samples. The signal from the carboxylic group (1715 cm^−1^) was not observed for DT0 and DT0_HCl. Only samples treated with HNO_3_ had COOH groups on the surface. Additionally, a weak signal from the hydrophilic group (1340 cm^−1^) was observed only for the carbons treated with HNO_3_. The absorption band appeared at 1548 cm^−1^ corresponding to the C=O stretching vibration [73]. This signal was present for all the samples, but for samples treated with HNO_3_, it was considerably higher. The presence of C=O groups on the surface of DT0 treated with HNO_3_ was enhanced. The intensity of signals attributed to functional groups containing oxygen was as follows: DT0_HCl_HNO_3_ > DT0_HNO_3_ > DT0_HCl ≈DT0. The broad peak located at about 1070 cm^−1^ related to the C–O stretching vibration of various functional groups [74] and a very small peak located about 1170 cm^−1^ related to C-C/C-O [75] were present on the surface of all samples. The FTIR results were consistent with the XPS results.

**Figure 5:**
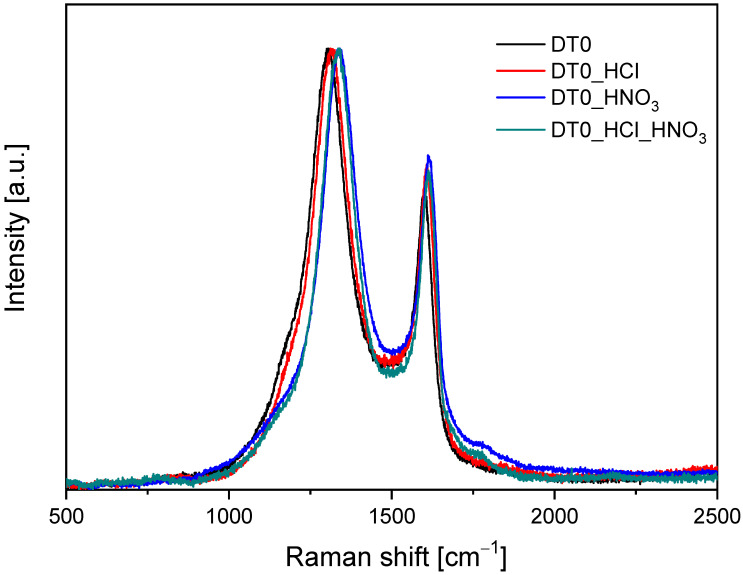
Raman spectra of samples.

The acid-treated DT0 carbons were characterized with Raman spectroscopy and are shown in Figure 5.

The G peak, centered around 1595 cm^−1^, can be assigned to the ordered carbon structure. G-band arises from the stretching of the C-C bond in graphitic materials and is common to all sp^2^ carbon systems. In the graphitic disorder of the carbon structure, the band around 1310 cm^−1^ named D was observed. The intensity ratio of the G-band and D-band (I_G_/I_D_) was used to evaluate the quality of carbon materials. The higher the I_G_/I_D_ value, the lower the graphitic disorder observed [76]. Table 5 shows the determined values of the I_G_/I_D_ ratios.

The values of I_G_/I_D_ for carbons were in the range of 0.65–0.72 and were similar to the previously reported for activated biocarbons [77]. The highest I_G_/I_D_ ratios were observed in the samples modified with acids in comparison to the unmodified DT0, which correspond to the better ordered structure in modified carbons.

**Figure 6:**
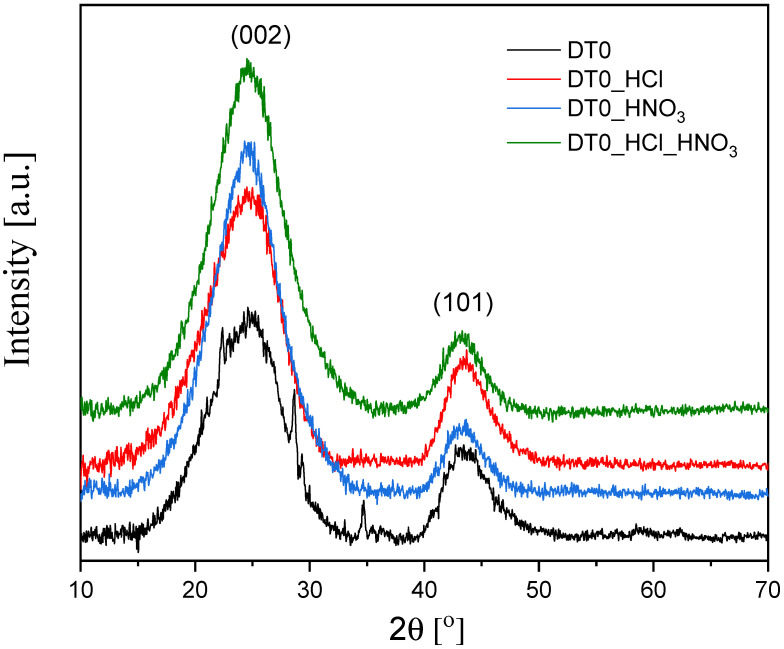
Diffraction patterns of DT0 carbons.

The graphitic structure of DT0 carbons was analyzed with the XRD method. The X-ray diffraction profiles for carbons are shown in Figure 6.

The diffractograms showed broad peaks and the absence of very sharp peaks. This indicates that the carbon was amorphous in nature [78]. The amorphous structure of the DT0 was identified by the peak at 2θ = 24° and peak at 2θ = 43°, which referred to the diffraction peaks (002) and (101) [79,80,81].

**Figure 7:**
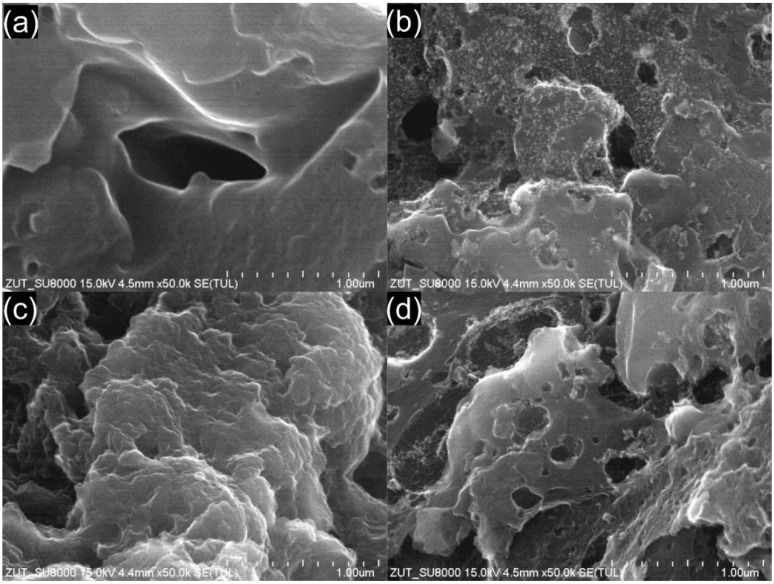
SEM images of activated carbon: (**a**) DT0, (**b**) DT0_HCl, (**c**) DT0_HNO_3_, and (**d**) DT0_HCl_HNO_3_.

The carbon morphology of studied samples at 50,000× magnifications is shown in Figure 7.

SEM images of the DT0 carbons showed that the adsorbent had texture with a heterogeneous surface and a variety of randomly distributed holes. The presence of holes of various diameters and irregular shapes was observed in DT0 HCl and DT0_HCl_HNO_3_. Contrary to that, a very smooth surface was identified for DT0. Similar shapes were observed for the other commercial activated carbons [82,83,84].

**Figure 8:**
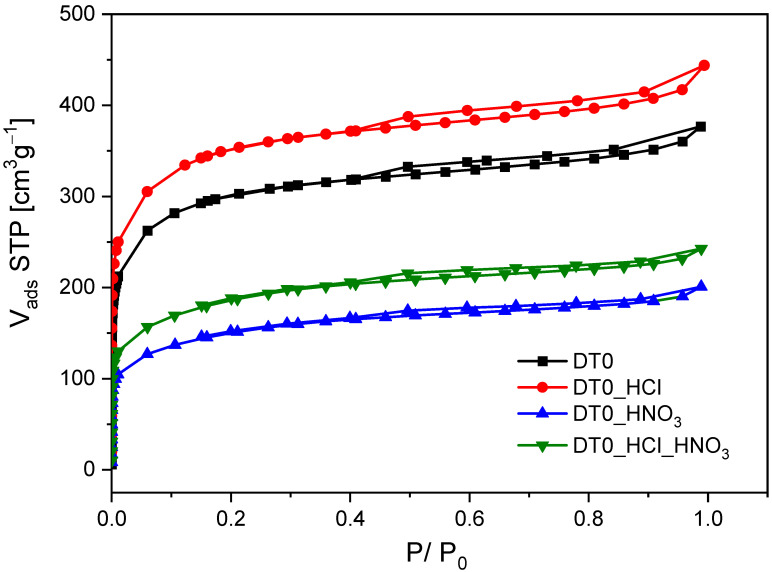
N_2_ adsorption–desorption isotherms of DT0 carbons.

The proposed methods of DT0 modification with inorganic acids influenced the textural properties. Figure 8 shows the nitrogen adsorption isotherms at −196 °C of the studied DT0 carbons.

The presented isotherms combine type I, which is characteristic of microporous materials, and type IV, which is characteristic of mesoporous materials [85]. Nitrogen adsorption increased very quickly at low P/P_0_ values, which is a characteristic feature of microporous materials. The formation of hysteresis loops at relative pressure higher than 0.4 indicated the multilayer adsorption process characterizing mesoporous structures. The hysteresis of catalysts was the type H4 associated with narrow slit pores, including pores in the micropore region. These are typical sorption isotherms for activated carbons [77,86].

**Figure 9:**
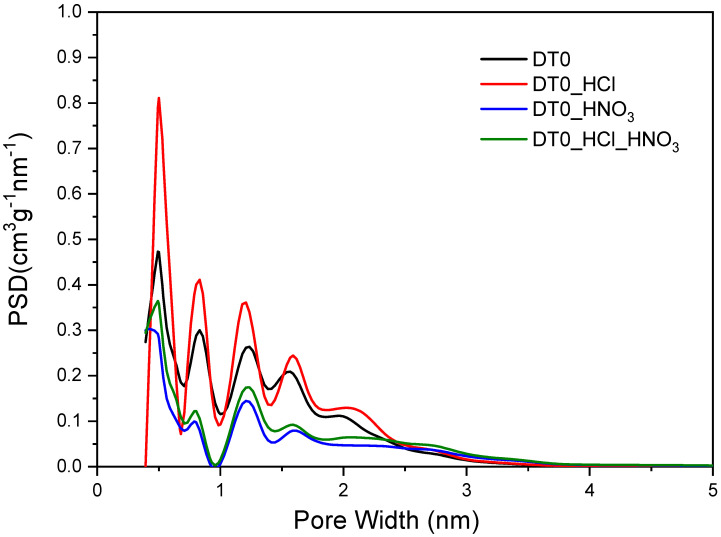
DFT pore size distribution of DT0 carbons.

Figure 9 shows the pore size distributions for the ACs determined by means of the N2 adsorption results at −196 °C using the density functional theory (DFT). Overall, it can be seen that the ACs have mainly micropores with a certain amount of mesopores.

It was stated that the acid-treated DT0 carbon samples showed significant differences in pore volume size compared to the pristine sample. In the case of DT0 carbon modified with nitric acid and the two-stage treatment with hydrochloric acid and nitric acid, a significant reduction in the pore volume in the range of 0.4–2.5 nm was observed. In contrast, treatment of DT0 with hydrochloric acid significantly increased the pore volume. This is because the treatment of AC with hydrochloric acid removes minerals, which in turn may increase the porosity. The DT0_HCl carbon had the best-developed micropores in the range of 0.4–2 nm. All the DT0 carbons contained mesopores with a size of 2–3.5 nm as well (Figure 9). Table 6 presents the textural parameters of DT0 carbons.

It was found that the treatment of DT0 carbon with HCl, HNO_3_, HCl, and HNO_3_ acids affected its porous structure to a greater or lesser extent. Treatment with HCl increased the surface area of the starting DT0 carbon from 1085 to 1267 m^2^/g and increased the total pore volume from 0.583 to 0.687 cm^3^/g. Thus, the increase of textural parameters in DT0_HCl may be caused by removing Na, K, and Ca salts by dissolving them in HCl, which promotes the development of the surface and pore structure.

It was evident from the EDX (Table 1) and XRF (Table 2) measurements that the minerals were dissolved with HCl and HNO_3_. Unfortunately, HNO_3_ simultaneously oxidized the carbon structure and damaged the micro- and mezopores. That is why only the S_BET_ of DT0_HCl increased (Table 6). The advantage of HNO_3_ treatment was the increase of oxygen-containing groups on the surface, especially COOH. The concentration of oxygen on the surface of DT0_HCl_HNO_3_ was similar to DT0_HNO_3_, but the S_BET_ and V_tot_ were higher than those of DT0_HNO_3_ because of the simultaneous action of both acids.

The effect of HNO_3_ had the greatest effect on reducing the specific surface area from 1085 to 532 m^2^/g, and the total carbon pore volume after modification was the smallest and amounted to 0.311 cm^3^/g. A fairly significant reduction in S_BET_ resulted from partial oxidation of carbon by strongly oxidizing nitric acid (V).

A significant reduction in textural parameters was also found in the case of carbon modified with HNO_3_ after previous modification with non-oxidizing HCl. With this two-step modification, the S_BET_ of DT0_HCl_HNO_3_ decreased from 1085 to 651 cm^3^/g and the total pore volume decreased to 0.375 cm^3^/g. However, this suggests that the double modification of carbon compared to the treatment of DT0 only with the oxidizing acid HNO_3_ had a more favorable effect on the development of porosity of the material. Similar conclusions leading to an increase in the porosity of carbons after their treatment with acids can be found in other studies [72,87]. There are also reports showing a decrease in the specific surface area of the carbons after acid treatment [88,89].

### 3.2. Alpha-Pinene Isomerization Process

**Figure 10:**
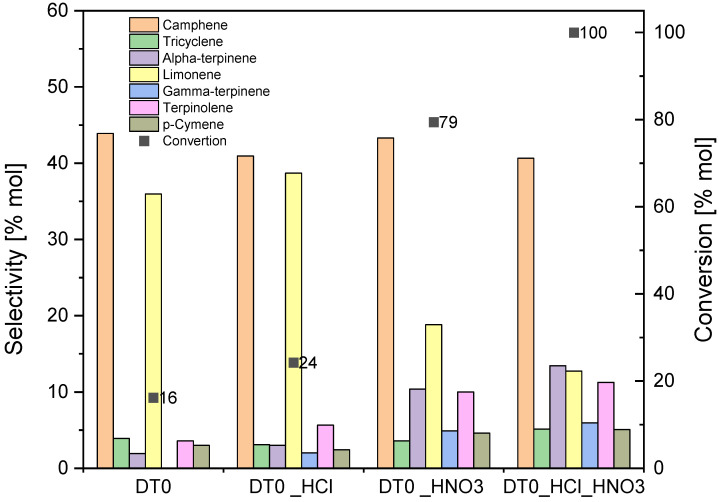
Comparison of the activity of DT0, DT0_HCl, DT0_HNO_3_, and DT0_HCl_HNO_3_ catalysts in the alpha-pinene isomerization process (applied conditions: temperature 160 °C, reaction time of 3 h, and the content of catalyst at 5 wt%).

In the first stage of catalytic tests, studies on alpha-pinene isomerization were carried out on DT0 carbon and on DT0_HCl, DT0_HNO_3_, and DT0_HCl_HNO_3_ carbons. The aim was to determine the most active catalyst for the process of α-pinene isomerization. At this stage, the following conditions were used in the tests: temperature 160 °C, the content of catalyst at 5 wt% (in relation to the amount of alpha-pinene), and reaction time of 3 h. Figure 10 shows the results of isomerization of alpha-pinene obtained in the study.

Figure 10 shows that the most active catalyst was DT0_HCl_HNO_3_. It allows for achieving the maximum conversion of alpha-pinene (100 mol%). The selectivities of the transformation to camphene and limonene on this catalyst were 41 and 13 mol%, respectively. The formation of alpha-terpinene (selectivity 13 mol%), gamma-terpinene (selectivity 6 mol%), terpinolene (selectivity 11 mol%), and p-cymene (selectivity 5 mol%) could also be observed on this porous material. Similar values of transformation selectivity were observed for the DT0_HNO_3_ catalyst, while the alpha-pinene conversion for the process carried out on this catalyst was lower by 21 mol%.

The results obtained on the other two catalysts are much worse, as the conversion on them reaches 16 mol% (DT0 catalyst) and 24 mol% (DT0_HCl catalyst). However, in the case of these two catalysts, attention is drawn to the very high selectivity of the transformation to limonene reaching the value of 36 mol% (DT0 catalyst) and 39 mol% (DT0_HCl catalyst), with the selectivity of transformation to camphene similar to that obtained on the previous two catalysts (44 mol% DT0 catalyst and 41 mol% DT0_HCl catalyst). On the other hand, the transformation selectivities to the remaining products are not significant for these two catalysts.

In the catalytic tests presented in this stage of the studies, the synergistic effect of HCl and HNO_3_ acids on DT0-activated carbon is also visible. It is visible in the fact that for the DTO_HCl_HNO_3_ sample, the selectivity of transformation to terpinolene increased compared to the unmodified DT0 sample and the DTO_HCl and DT0_HNO_3_ samples (more than twofold increase). A similar effect was seen with p-cymene, gamma-terpinene, and alpha-terpinene. The synergistic effect of HCl and HNO_3_ on the DT0 carbon simultaneously reduced the selectivity of transformation to limonene as compared to the unmodified DT0 sample (by about 20 mol%).

The comparison of the results presented in Table 3 (tests using the XPS method) shows that the DT0_HNO_3_ and DT0_HCl_HNO_3_ catalysts differ significantly (almost 3 times) from the other two catalysts in the content of carboxyl groups (COOH group). They also have slightly more C-OH groups (DT0 and DT0_HCl with 12.6 and 12.3, and DT0_HNO_3_ and DT0_HCl_HNO_3_ with 13.7 and 14.2, respectively). It should also be noted that, in the case of DT0_HNO_3_ and DT0_HCl_HNO_3_ carbon materials, no keto-enolic groups were observed. This increased content of COOH and C-OH groups in the DT0_HNO_3_ and DT0_HCl_HNO_3_ carbon materials and the lack of keto-enolic groups in them may be the reason for the greater activity of DT0_HNO_3_ and DT0_HCl_HNO_3_ in the alpha-pinene isomerization process. It seems more advantageous to wash the DT0 carbon with the aqueous solution containing both nitric acid and hydrochloric acid, and not with the aqueous solution of the nitric acid alone.

Based on the research results presented at this stage, the DT0_HCl_HNO_3_ catalyst was considered to be the most active.

**Figure 11:**
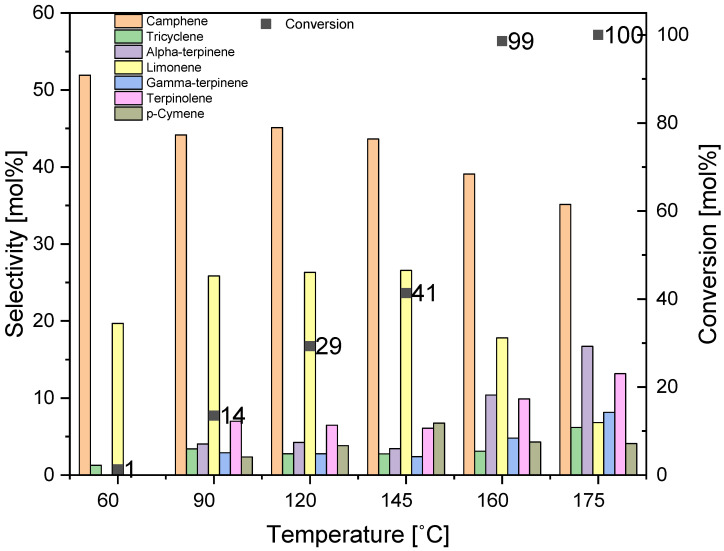
Influence of temperature on alpha-pinene isomerization process for DT0_HCl_ HNO_3_ catalyst (applied conditions: content of catalyst at 5 wt% and reaction time of 3 h).

The research on the effect of temperature on the course of alpha-pinene isomerization on the DT0_HCl_HNO_3_ material is shown in Figure 11. The tests were carried out at the following temperatures: 60, 90, 120, 145, 160, and 175 °C, for 3 h and with catalyst content of 5 wt%.

The comparison of the results obtained at the two lowest temperatures (60 and 90 °C) shows that these temperatures are too low to carry out the alpha-pinene isomerization process, as the conversion of this raw material is only 1 and 14 mol%. In addition, the process temperature increasing from 60 to 90 °C resulted in a decrease of the selectivity of transformation to camphene (from 52 mol% to 44 mol%). The further increase of temperature to 145 °C did not cause any significant changes in the selectivity of the transformation to camphene. Comparing the results obtained for the two lowest temperatures, it can also be noticed that increase of temperature increases the selectivity of transformation to limonene (from 20 to 26 mol%), terpinolene (from 0 to 7 mol%), alpha-terpinene (from 0 to 4 mol%), gamma-terpinene (0 to 3 mol%), tricyclene (1 to 3 mol%), and p-cymene (0 to 2 mol%). The highest alpha-pinene conversions were obtained by carrying out the isomerization process at temperatures of 120 and 145 °C at 29 and 41 mol%, respectively. The highest alpha-pinene conversions were obtained by carrying out the isomerization process at temperatures of 160 and 175 °C (99 and 100 mol%, respectively). At these temperatures, the highest selectivity of transformation to several products was also obtained: tricyclene—3 and 6 mol%, alpha-terpinene—10 and 17 mol%, gamma-terpinene—5 and 8 mol%, and terpinolene—10 and 13 mol%. The highest selectivity of transformation to limonene was obtained at temperatures of 120 and 145 °C, then its value decreased (to 7 mol%). As the process temperature increased, an increase in the selectivity of the transformation to the dehydrogenation product (p-cymene) was also observed, and at the highest temperatures, it was about 4–7 mol%.

**Figure 12:**
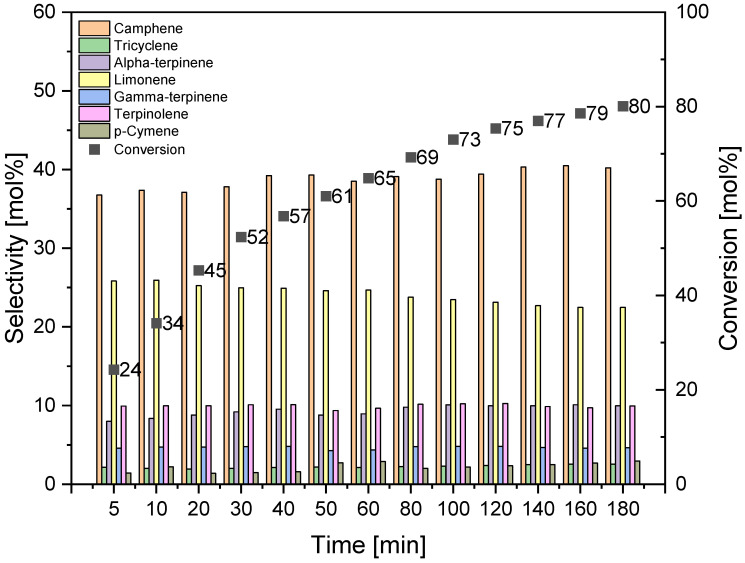
Effect of reaction time on alpha-pinene isomerization on DT0_ HCl _NO_3_ catalyst at 145 °C (catalyst amount 5 wt%).

**Figure 13:**
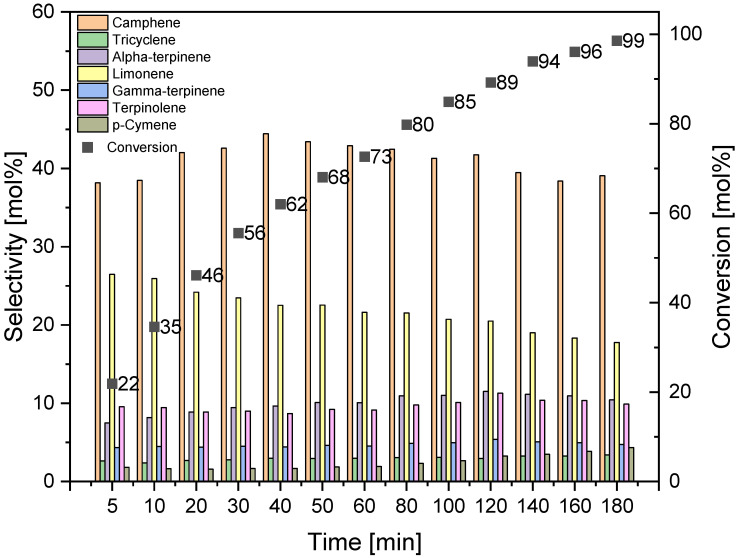
Effect of reaction time on alpha-pinene isomerization on DT0_ HCl _NO_3_ catalyst at 160 °C (catalyst amount 5 wt%).

**Figure 14:**
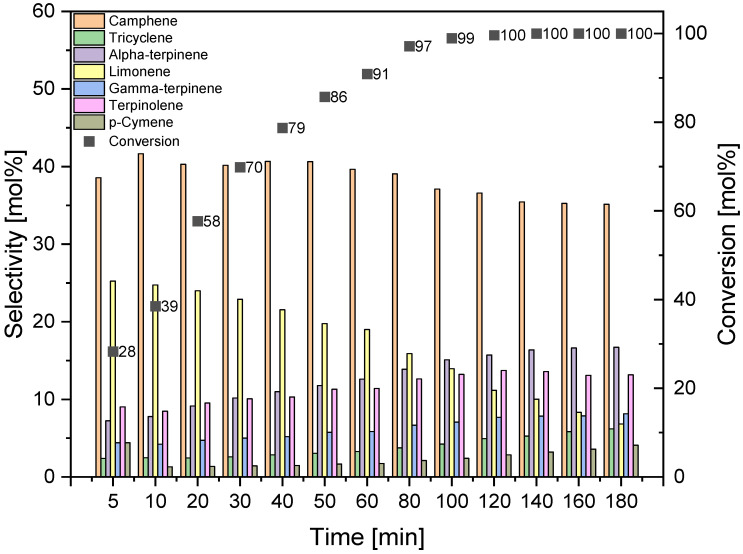
Effect of reaction time on alpha-pinene isomerization on DT0_ HCl _NO_3_ catalyst at 175 °C (catalyst amount 5 wt%).

To study the effect of reaction time on alpha-pinene isomerization, three temperatures were selected at which the highest values of alpha-pinene conversion were obtained: 145, 160, and 175 °C. The tests were carried out with DT0_HCl_HNO_3_ catalyst in the reaction mixture of 5 wt% and in the range of reaction times from 5 to 180 min. The results are presented in Figure 12, Figure 13 and Figure 14.

A comparison of the results obtained at the three tested temperatures shows that the alpha-pinene conversion of about 100 mol% can be achieved by carrying out isomerization at the two highest temperatures, while for the temperature of 160 °C the time required to achieve such a high conversion is 180 min, and for the temperature of 175 °C the time required is 100 min, which is significantly shorter. The maximum selectivities of the transformation to camphene obtained at the three tested temperatures are similar and amount to 41–44 mol%. The highest selectivity of tricyclene was noted for the process carried out at the temperature of 175 °C and for the reaction time of 160–180 min (6 mol%). At the same temperature of 175 °C and for the same reaction time, the highest selectivity of alpha-terpinene (about 17 mol%), gamma-terpinene (about 8 mol%), terpinolene (about 13 mol%), and p-cymene (4 mol%) was also observed. Figure 12, Figure 13 and Figure 14 also show that the highest selectivity of camphene (40–44 mol%) can be achieved by carrying out the isomerization process: longer than 40 min (the most advantageous are 40–50 min) at 145 °C, for 40–50 min at 160 °C, or for 10–50 min at 175°C. The comparison of the results for the selectivity of transformation to limonene shows that the selectivity of this compound decreases with increasing the reaction time and increasing the temperature, and the highest selectivity of the transformation to this compound (about 24–25 mol%) is obtained for short reaction times: for the temperature of 145 °C for a reaction time up to 30 min, for temperatures of 160 °C and 175 °C for a time reaction time not exceeding 10 min.

### 3.3. Kinetic Studies

The comprehensive kinetic studies of α-pinene isomerization at a temperature of 145 °C, 160 °C, and 175 °C over DT0_HCl_HNO_3_ were performed for several orders, using the following equations.

For first order:

(1) $$-\frac{dC_{\alpha \text{-}pinene}}{dt}=kC_{\alpha \text{-}pinene},$$

For orders different from one:

(2) $$\frac{C_{\alpha \text{-}pinene}^{1-n}-C_{\alpha \text{-}pinene_{0}}^{1-n}}{n-1}=\mathrm{kt},$$

where *C_α-pinene_* is the α-pinene concentration, t is the reaction time, and k is the reaction rate constant.

Kinetic parameters for the kinetic equations were determined using a parameter estimation software. The highest regression coefficients were obtained for the first-order reaction for α-pinene isomerization in all three reaction temperatures. Therefore, the consumption rate of α-pinene followed the first-order kinetics. This reaction order matches our previous results achieved for instance for α-pinene isomerization over Ti_3_C_2_ and ex-Ti_3_C_2_ [66] over clinoptilolite (modified with 0.1 M H_2_SO_4_—CLIN 0.1) [63]. Moreover, similar results were also reported by other authors, namely, Ünveren et al. [90] and Allahverdiev et al. [91].

The calculated reaction rate coefficients for the first-order reaction are compiled in Table 7.

The highest value of the reaction rate constant was calculated at 175 °C (k = 2.74 h^−1^). This value is nearly six times higher than the calculated constant at 140 °C (k = 0.47 h^−1^).

Dependence of these kinetic constants as a function of temperature strongly supports the assumption of the first-order kinetics. Temperature dependence of the first-order kinetic constant obeyed Arrhenius dependence with the global activation energy equal to 91.5 kJ/mol. Calculated activation energy matches results achieved for typical values of activation energy for a-pinene isomerization (for example 80.9 kJ/mol [17]).

## 4. Conclusions

The modification of activated carbons with acids is very beneficial compared with plasma. The method presented here is considerably cheaper. The plasma oxidized only the outside surface of the grains. The acid treatment oxidized the inside and outside surface and also removed the minerals blocking the pores.

The modification of carbon material DT0 with acid treatment showed that it is more advantageous to wash DT0 carbon with the aqueous solution containing both nitric acid and hydrochloric acid, and not with the aqueous solution of the nitric acid alone. The least preferred method is washing of DT0 carbon with the solution of hydrochloric acid alone. DT0 carbons washed with the aqueous solution of nitric acid with hydrochloric acid and with the aqueous solution of nitric acid alone showed the presence of the increased amount of COOH and C-OH groups, which is most likely the reason for their increased activity in the alpha-pinene isomerization process compared to pure DT0 carbon and DT0 carbon treated with the aqueous solution of hydrochloric acid. Moreover, the activities of the DT0_HNO_3_ and DT0_HCl_HNO_3_ carbons may also be influenced by the lack of keto-enolic groups in these samples.

## Figures and Tables

**Table 1:** The elemental analysis based on EDX measurements.

	DT0 [wt%]	DT0_HCl [wt%]	DT0_HNO_3_ [wt%]	DT0_HCl_HNO_3_ [wt%]
C	51	66.1	48.5	62.8
O	33.9	29.9	46.5	34.5
Na	0.9			
Al	3.9	0.3	0.5	0.3
Si	5.1	3.7	4.5	2.4
K	2.9			
Ca	0.8			
Fe	1.5			

**Table 2:** The elemental analysis based on XRF measurements.

	DT0 [wt%]	DT0_HCl [wt%]	DT0_HNO_3_ [wt%]	DT0_HCl_HNO_3_ [wt%]
Al	2.1			
Si	4.1	2.7	3.1	2.3
K	2.9			
Ca	1.2		0.1	
Fe	1.7	0.3	0.2	0.1

**Table 3:** The content of C 1s components expressed as atomic concentration.

Assignment	DT0	DT0_HCl	DT0_HNO_3_	DT0_HCl_HNO_3_
C	63.1	63.1	60.5	61.0
C-OH	12.6	12.3	13.7	14.2
keto-enolic	4.1	2.8	0.0	0.0
C=O	7.1	7.5	7.8	7.8
COOH	2.6	2.1	6.4	6.3
satellite	10.7	12.1	11.7	10.8

**Table 4:** Elemental content of the sample surfaces.

		%at		
	C1s	O1s	Si2p	Ca2p
DT0	68.2	26.4	4.8	0.6
DT0_HCl	56.6	32.8	10.3	0.3
DT0_HNO_3_	59.2	34.6	6.1	0.1
DT0_HCl_HNO_3_	59.7	34.2	6.0	0.1

**Table 5:** Values of the I_G_/I_D_ ratios of activated carbons modified with HCl and HNO_3_.

Sample	I_G_/I_D_
DT0	0.65
DT0_HCl	0.70
DT0_HNO_3_	0.71
DT0_HCl_HNO_3_	0.70

**Table 6:** Textural parameters of DT0 carbons.

Carbon	S_BET_ [m^2^/g]	V_tot_ [cm^3^/g]	V_mic_ [cm^3^/g]
DT0	1085	0.583	0.349
DT0_HCl	1267	0.687	0.407
DT0_HNO_3_	532	0.311	0.160
DT0_HCl_HNO_3_	651	0.375	0.197

**Table 7:** The a-pinene isomerization kinetic parameters.

Temperature [°C]	k [h^−1^]	R^2^
145	0.47	0.8875
160	1.22	0.9741
175	2.74	0.9914

## Data Availability

The data presented in this study are available on request from the corresponding author.
